# United States multi-sector land use and land cover base maps to support human and Earth system models

**DOI:** 10.1038/s41597-025-04713-6

**Published:** 2025-03-19

**Authors:** Jay Oliver, Ryan A. McManamay

**Affiliations:** Department of Environmental Science, Baylor University, Waco, TX 76798 USA

**Keywords:** Environmental impact, Climate-change adaptation

## Abstract

Earth System Models (ESMs) require current and future projections of land use and landcover change (LULC) to simulate land-atmospheric interactions and global biogeochemical cycles. Among the most utilized land systems in ESMs are the Community Land Model (CLM) and the Land-Use Harmonization 2 (LUH2) products. Regional studies also use these products by extending coarse projections to finer resolutions via downscaling or by using multisector dynamic (MSD) models. One such MSD model is the Global Change Analysis Model (GCAM), which has its own independent land module, but often relies on CLM or LUH2 as spatial inputs for its base years. However, this requires harmonization of thematically incongruent land systems at multiple spatial resolutions, leading to uncertainty and error propagation. To resolve these issues, we develop a thematically consistent LULC system for the conterminous United States adaptable to multiple MSD frameworks to support research at a regional level. Using empirically derived spatial products, we developed a series of base maps for multiple contemporary years of observation at a 30-m resolution that support flexibility and interchangeability amongst LUH2, CLM, and GCAM classification systems.

## Background & Summary

Earth System Models (ESM) portray current and future land use and landcover change (LULCC) dynamics to understand land-atmospheric interactions and their implications on global climate and biochemical cycles^1^. Multiple ESMs are represented in the Coupled Model Intercomparison Project (CMIP)^2^, such as Community Earth System Model (CESM)^3^. Among the most utilized and documented land modeling systems (lms) within CMIP are the Community Land Model (CLM)^4,5^, the land model for CESM, and the Global Land-Use Model 2 (GLM2), that latter model underlying the Land-Use Harmonization 2 (LUH2)^6^ dataset. These lms are essential within their respective ESM frameworks since they support historical and future dynamic projections of landcover components, yet they have also been used independently to support numerous LULCC studies when needed^7,8^. Both LUH2 and CLM have been used for a multitude of numerical experiments, including the implications of LULCC on carbon budgets^9–17^, biogeochemical cycles^18–20^, land surface processes^21,22^; agricultural sector changes^23,24^; and lastly, inter-relational dynamics between land and other physical processes, including climate^25–27^, impacts of extremes (drought and wildfire)^28,29^, hydrologic processes^30^, and heat fluxes^31^.

Despite the widespread use of these products, characterizing future LULCC and implications for associated processes have been limited by the absence of standardized LULCC products^21,32^ and inconsistencies among products^33^ or between products and empirical observations^21,34^. These differences become even more problematic when attempting to characterize land uses that reflect human activities, which have lasting impacts on global processes^1^ yet are evolving and highly uncertain. Projections of the dynamic future roles of human agents within the Earth’s System can be modeled using Multi-sector Dynamics (MSD) model frameworks^35^, which depict alternative scenarios of future land states based on socioeconomic drivers^36–39^. One such example is the Global Change Assessment Model (GCAM), which models human and natural system behaviors and interactions between energy, water, land, climate, and economic systems at several scales^39^. Specifically, GCAM develops land allocations at a sub-regional level^36^. Intercomparisons and cross-validation of LULCC projections between GCAM, LUH2, and CLM are valuable to support integrative and inter-disciplinary efforts, such as CMIP series. However, integration and inter-comparisons among CLM, LUH2, and GCAM land system classifications are challenging because these systems are not entirely compatible^3,4,39^. CLM classifications are based on 79 different “plant functional types” (PFTs) along with three constant land types for Ice, Water, Wetlands and an Urban canopy model^3^. Similarly, LUH2 relies on 12 “states” along with a supporting land type for Ice and Water^6^, whereas GCAM uses 39 “landcover types” (GLTs)^33,40^. Additionally, the native spatiotemporal resolutions of these land system products are highly divergent.

Another complication is that as ESMs become more complex, they require integration and coupling of multiple model frameworks that leverage each other’s strengths; however, this creates harmonization limitations. For example, although GCAM resolves land dynamics at a sub-regional level, it lacks a spatially explicit and contiguous gridded representation (i.e., rasterized) of the Earth’s land surface^33^. To extend GCAM allocations to the land surface, the Demeter model was developed to disaggregate and downscale land allocations to a local resolution; this approach has supported multiple regional model efforts^41–43^. However, Demeter requires a pre-existing base map of landcover as an initialization for the model, and most commonly relies on CLM for this task^40^. This requires harmonization between CLM PFTs and GCAM GLTs, which are not entirely compatible. Chen *et al*.^33^ developed a GCAM-Demeter product using CLM inputs as a base map and then compared the modeled outputs with LUH2, as a separate model validation, showing highly divergent results between GCAM-Demeter and LUH2 projections.

Apart from harmonization of thematically divergent land classes, CLM and LUH2 products vary considerably in spatial granularity and data format making empirical validations difficult. While the resolutions of CLM (0.05°) and LUH2 (0.25°) are appropriate for global and inter-regional studies^6,44^, these resolutions are far too coarse to support localized land modeling efforts, such as high-resolution hydrologic modeling^34,45–49^. Additionally, CLM and LUH2 are fractionated, rather than mutually exclusive, land class products, which despite accommodating for uncertainty in the spatial allocations of land types, these data structures can be gross representations of realistic land cover patterns, leading to inaccuracies in modeling simulations^5,21,34,45,46^. In contrast, many of the contemporary empirical land cover products are derived from high-resolution (<= 30m) satellite observations, where multispectral bands can be used to develop mutually exclusive, rather than fractionated, land cover products, such as the National Land Cover Dataset (NLCD)^50^. Indeed, high-resolution landcover products are advantageous to not only depict and differentiate amongst human alternation of land surfaces^51,52^, but also serve as a more accurate, empirically based product to support validation and regional modeling within the MSD modeling communities^35,53^.

Ultimately, a standardized, consistent, and empirically based LULCC system is needed to reconcile many of the issues presented above. Furthermore, such a system provides a valuable resource to couple ESMs with regional MSD studies, especially for large regions such as the United States. To maximize versatility and flexibility of use across multiple applications, we envisioned an ideal landcover system would have several attributes, including: 1) a common source of LULC spatial information to support interchangeability, harmonization, and intercomparisons among divergent land modeling systems and support up- or down-scaling efforts between global and regional scales, 2) a series of standardized basemaps as initial conditions to support future LULCC model projections for regional applications, 3) a hierarchical structure to support flexible re-organization or re-grouping of classes to match thematic differences amongst land classification systems, 4) use of best available empirical observations for mapping land uses and land cover to ensure consistency among products and standardized validation, and 5) a high resolution (~30 m) to support modeling of local hydrologic and biogeochemical processes.

Herein, we aimed to meet this need for US regional studies by developing a multisector land classification system for the Continental United States (CONUS) extendable to multiple MSD frameworks. We focused on developing a series of Multisector Dynamic (MSD) base maps relevant to GCAM, CLM, and GLM-LUH2 at a 30-m resolution since multiple US-based empirical products were already available at that resolution. Specifically, the USGS NLCD^54^ and the USDA Cropland Data Layer (CDL)^55^, along with other available geospatial products (e.g., MODIS), were critical to developing such a product. The goal of developing a series of MSD base maps was to produce a hierarchical land classification (lc) system that was flexible enough to support the most resolved classification system needed, but also to support selection of coarsened land class groupings relevant to multiple human-earth system modeling frameworks. As a validation, we compare our empirically base maps to CLM and LUH2 products to examine associations or incompatibilities.

## Methods

### Overview of the multisector dynamic (MSD) base map

**Fig. 1:**
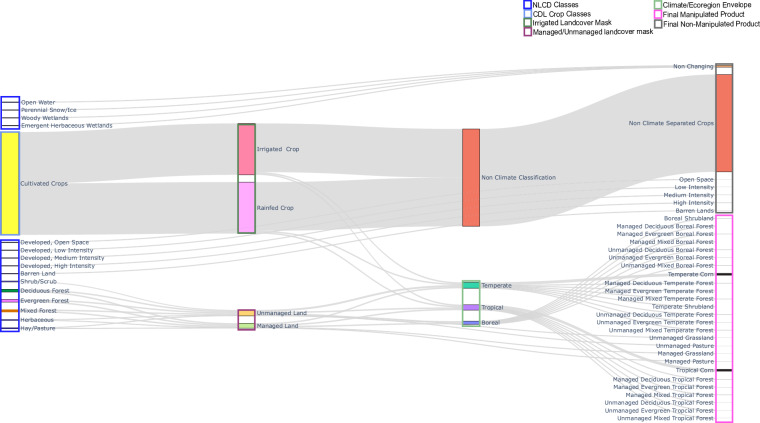
Flow-chart showing empirical, high-resolution land maps (including National Land Cover Dataset (NLCD) and Crop Data Layers (CDL) layers) on the far left and the processing steps taken to develop complete inventory of MSD layers (far right) compatible with GCAM, CLM, and LUH2. Some of the NLCD layers were directly mapped to the final MSD land types (Non-manipulated product). CDL Crop layers were first extracted from the NLCD cultivated crops class and were overlayed with an irrigated land mask^53^ to differentiate between irrigated and rainfed crops. The PADUS3^54^ dataset was used to differentiate between managed and unmanaged land. Lastly, a climate and ecoregion envelope^58–62^ was developed to differentiate between temperate tropical and boreal landcover.

Development of the MSD base maps followed a sequential process. To inform our land classification typology, we first developed a list of all the land classes available through high-resolution satellite derived products in the US. This consisted of compiling landcover and land use classes from the National Land Cover Dataset (NLCD)^50^ and from the United States Department of Agriculture Crop Data Layer (CDL)^56^ (https://www.nass.usda.gov/Research_and_Science/Cropland/SARS1a.php), which collectively represent the most widely used, empirically derived (via satellite), and routinely updated and maintained LULCC observation datasets in the US. These datasets form the initial foundation of accurate spatial extents of various LULC classes used to create our final MSD base map (Fig. 1). We then compiled a list of all land classes found within GCAM, LUH2, and CLM and compared these classes with those from NLCD and CDL. This provided a ‘master list’ of land classes, which served as a template to establish a hierarchical land typology that could support a thematically diverse LULC base map for the US while also being inter-changeable for use among multiple land modeling systems. We noted compatibilities between classes where direct translation was possible and cases where more spatial information on land use (e.g., irrigation or land protection) was needed for class associations. The additional sources of information used in our analysis are described more fully below. We then developed a workflow to translate land information from NLCD and CDL into all land classes in the master list by aggregating, refining, or in some instances, leveraging other land use information to differentiate management structures for those land types (Fig. 1). The most thematically resolved individual land cover types were then mapped and combined to produce the final and most detailed MSD base maps for multiple years based on data availability. In turn, the land classes within these detailed maps could then be reorganized to produce base maps specific to CLM, LUH2, and GCAM.

### Compiling empirical landcover products and pre-processing

After comparing all land classifications among the human-earth system land model frameworks (GCAM, LUH2, CLM) with the high-resolution empirical products (NLCD, CDL), we developed a final hierarchical land system comprised of 259 classes (Supplemental Material 1). The MSD hierarchical land classification system can be stand alone or regrouped into GCAM Landcover Types (GMLTs) (Table 1), LUH2 based MSD Landcover Types (LMLTs) (Table 2), and CLM based MSD Landcover Types (CMLT) (Tables 3, 4). For example, the MSD includes four urban classes, which were taken from the NLCD (open, low, medium, and high intensity development). These four classes can be aggregated to mirror the singular urban class represented in GCAM, CLM, or LUH2.

**Table 1:** GCAM land classes and their associated MSD land classes for the CONUS.

GCAM Class	GCAM Identifier	GMLT Expanded Values	MSD Data Layers
Corn: Irrigated	1	1	1–3, 7, 8, 9
Corn: rainfed	2	2	4–6, 10, 11, 12
Wheat: irrigated	3	3	13–15, 16
Wheat: rainfed	4	4	17–19, 20
Rice: irrigated	5	5	21
Rice: rainfed	6	6	22
Root tuber: irrigated	7	7	23–25
Root tuber: rainfed	8	8	26–28
Oil Crop: irrigated	9	9	29–35
Oil crop: rainfed	10	10	36–42
Sugar crop: irrigated	11	11	43–45
Sugar crop: rainfed	12	12	46
Other grain: irrigated	13	13	47–53
Other grain: rainfed	14	14	54–60
Fiber crop: irrigated	15	15	61
Fiber crop: rainfed	16	16	62
Fodder grass: irrigated	17	17	63
Fodder grass: rainfed	18	18	64
Fodder herb: irrigated	19	19	65–68
Fodder herb: rainfed	20	20	69–72
Biomass grass: irrigated	21	21	73, 74
Biomass grass: rainfed	22	22	75, 76
Misc-crop: irrigated	25	25	77–150
Misc-crop: rainfed	26	26	151- 224
Other arable land	27	27	225, 226
Managed Pasture	30	30	227, 253
Unmanaged Pasture	31	31	228
Urban	32	40	229
		41	230
		42	231
		43	232
Managed Forest	34	34	233–241
Unmanaged Forest	35	35	242–250
Shrubland	36	36	251, 252
Grassland	37	37	254
Rock, Ice, Desert	39	39	255, 256
Open Water	—	44	257
Woody Wetlands	—	45	258
Emergent Herbaceous Wetlands	—	46	259

Three GCAM class classes that are not modeled in GCAM include “Open Water”, “Woody Wetlands” and “Emergent Herbaceous Wetlands”, but are mapped within the NLCD. GMLT expanded values include four urban types based on the NLCD (Developed, Open Space, Developed Low Intensity, Developed Medium Intensity, Developed High Intensity) and Non-Changing Landcover types, also found in the NLCD (Open Water, Woody Wetlands, Emergent Herbaceous Wetlands). Respective MSD land values that were combined into their respective GCAM and GMLT values. MSD class descriptions can be found in Table S1.

**Table 2:** LUH2 land states and their associated MSD land classes for the CONUS.

LMLT Expanded Values	LMLT Identifier	MSD Layer Classification
C3_Annual_Crops	1	13–23, 25, 26, 28, 30–34, 37–41, 43, 45, 47–50, 52–57, 59–64, 67, 68, 71–73, 75, 78, 81, 83, 84, 85, 87, 88, 91, 100, 101, 108, 111, 112, 114, 120, 123, 125, 126, 127–134, 136, 138, 143–148, 152, 155, 157, 158, 159, 161, 162, 165, 174, 175, 182, 185, 186, 188, 194, 197, 199–208, 210, 212, 217–222
C3_Nitrogen_Fixing_Crops	2	29, 36, 66, 70, 77, 80, 82, 89, 90, 139, 140, 141, 150, 151, 154, 156, 163, 164, 213, 214, 215, 224
C3_Perennial_Crops	3	24, 27, 35, 42, 74, 76, 79, 86, 92 – 110, 113, 115, 116, 117- 119, 121, 122, 124, 142, 149, 153, 160, 166–184, 187, 189–193, 195, 196, 198, 216, 223
C4_Annual_Crops	4	1–12, 51, 58, 135, 137, 209, 211
C4_Perennial_Crops	5	44, 46, 65, 69
Managed_Pasture	6	227, 253
Forested	7	233–250, 258
Non_Forested	8	225, 226, 256,259
Rangeland	9	228, 251, 252, 254
Urban	10	229–232
Ice_Water	11	255, 257

Primary and secondary stages of forest land classes were combined into a single forested class for both the forested and non-forested states. MSD class descriptions can be found in Table S1.

**Table 3:** CLM Plant Functional Types (PFTs) found within the CONUS and their respective MSD classes.

PFT value	PFT Name	MSD class values
PFT_1	Non-Vegetated	256
PFT_2	Needleleaf evergreen tree-temperate	237, 240, 246, 249
PFT_6	Broadleaf evergreen tree-temperate	
PFT_3	Needleleaf evergreen tree-boreal	236, 239, 245, 248, 257
PFT_7	Broadleaf deciduous tree-tropical	235, 238, 241, 244, 247, 250
PFT_8	Broadleaf deciduous tree-temperate	234, 243
PFT_9	Broadleaf deciduous tree-boreal	233, 242
PFT_10	Broadleaf evergreen shrub-temperate	252
PFT_11	Broadleaf deciduous shrub-temperate	
PFT_12	Broadleaf deciduous shrub–boreal	251
PFT_13	C3 Arctic	227, 228, 253, 254
PFT_14	C3 Grass	
PFT_15	C4 Grass	225, 258
PFT_16	C3 unmanaged rainfed crop	17, 20, 28, 38, 39, 41, 42, 55, 57, 59, 60, 70–72, 75, 152, 153, 155, 157–159, 160, 161, 165–172, 174, 175, 177–186, 188, 189, 191, 192, 194–196, 197, 198, 200–208, 216–223, 226
PFT_17	C3 unmanaged irrigated crop	13, 16, 25, 31, 32, 34, 35, 48, 50, 52, 53, 66–68, 73, 78, 79, 81, 83–87, 91–101, 103–112, 114–118, 120–124, 126–134, 142–149, 190
PFT_18	Rainfed temperate corn	4–6, 215
PFT_19	Irrigated Temperate Corn	1–3, 141
PFT_20	Rainfed spring wheat	18
PFT_21	Irrigated spring wheat	14
PFT_22	Rainfed winter wheat	19, 154, 199, 210, 212
PFT_23	Irrigated winter wheat	15, 80, 125, 136, 138
PFT_24	Rainfed temperate soybean	36, 213, 214
PFT_25	Irrigated temperate soybean	29, 139, 140
PFT_26	Rainfed barley	54, 209, 211, 224
PFT_27	Irrigated barley	47, 135, 137, 150
PFT_30	Rainfed rye	56
PFT_31	Irrigated rye	49
PFT_36	Rainfed citrus	176, 187, 193
PFT_37	Irrigated citrus	102, 113, 119
PFT_42	Rainfed cotton	62
PFT_43	Irrigated cotton	61
PFT_46	Rainfed foddergrass	63
PFT_47	Irrigated foddergrass	64

MSD class codes are found in Table S1. CLM classes were grouped in situations where we were unable to spatially distinguish different types. PFT 2 and PFT 6 were both classified into a single temperate evergreen tree class, PFT 10 and PFT 11 were both classified into a temperate shrub class, and PFT 13 and PFT 14 were both classified into a C3 grass class. MSD class descriptions can be found in Table S1.

**Table 4:** Table 3 continued.

PFT value	PFT Name	MSD Values
PFT_48	Rainfed grapes	173
PFT_49	Irrigated grapes	99
PFT_50	Rainfed groundnuts	151
PFT_51	Irrigated groundnuts	77
PFT_52	Rainfed millet	58
PFT_53	Irrigated millet	51
PFT_56	Rainfed potatoes	26, 27
PFT_57	irrigated potatoes	23, 24
PFT_58	Rainfed pulses	156, 162, 163, 164
PFT_59	Irrigated pulses	82, 88, 89, 90
PFT_60	Rainfed rapeseed	40
PFT_61	Irrigated rapeseed	33
PFT_62	Rainfed rice	22
PFT_63	Irrigated rice	21
PFT_64	Rainfed sorghum	69
PFT_65	Irrigated sorghum	65
PFT_66	Rainfed sugarbeet	46
PFT_67	Irrigated sugarbeet	45
PFT_68	Rainfed sugarcane	44
PFT_69	Irrigated sugarcane	43
PFT_70	Rainfed sunflower	37
PFT_71	Irrigated sunflower	30
PFT_74	Rainfed switchgrass	76
PFT_75	Irrigated switchgrass	74
PFT_76	Rainfed tropical corn	10–12
PFT_77	Irrigated tropical corn	7–9
PFT_78	Rainfed tropical soybean	-
PFT_79	Irrigated tropical soybean	-
lake	Open water	257
glacier	Glacier, perennial snow	255
urban	urban	229–232
lanwat	wetlands	257, 258

To ensure thematic consistency and crisp hierarchical relationships among different classification systems, we mapped all products to visually examine spatial overlap among land classes from separate modeling systems. Landcover from LUH2 was obtained from the LUH2 website (https://luh.umd.edu/data.shtml)^6^, whereas landcover from CLM was obtained from the CESM website, specifically the list of raw land products (https://svn-ccsm-inputdata.cgd.ucar.edu/trunk/inputdata/lnd/clm2/rawdata/)^57^. We obtained empirically derived spatial datasets that would give us a detailed view of landcover diversity within the United States at a 30-m resolution. Figure 1 displays our basic workflow starting with land classes represented by the empirical observation datasets and how those were reorganized, filtered, or processed with other sources of information (e.g., irrigation, land protection, and climate region) to attain the final detailed classification of all MSD land classes. The USDA CDL represented only crop-specific layers, whereas for all other layers, we used the NLCD. Empirical sources of information were limited to years where such data (i.e., NLCD and CDL) were available and overlapped at the time of our analysis, which included 2008, 2011, 2013, 2016, and 2019 (note that more recent years are available).

### Class-specific processing and refinement

Prior to any processing with the actual layers, we merged the CDL with the NLCD by reclassifying NLCD cultivated crop and pasture/hay land classes with crop-specific CDL class values to support the development of a spatially comprehensive and mutually exclusive land classification system. This was particularly important for crop classes within the CDL because croplands were the most diverse layers. To ensure compatibility amongst crop classifications from the CDL, GCAM, LUH2, and CLM, we relied on translations between FAO and GCAM commodities^58^. All CDL layers (including *fallow/idle cropland)* were placed into a category based on their respective FAO descriptor^58^. A special case was required for developing the “*other arable land”* class. For this step, we combined *fallow/idle cropland* from the CDL with *cultivated crops* from NLCD to include all potential crops that were not accounted for in other classes. We then developed a mosaic raster combining all crop classes within the CDL for a given year and overlaid the mosaic with the corresponding NLCD layer. This mosaic resulted in mutually exclusive, non-overlapping NLCD and CDL pixels that would be used to extract the remaining landcover types to support reclassification into the other land modeling systems (GCAM, LUH2, and CLM).

### Rainfed and irrigated cropland

Because the CDL provides no specificity of irrigation at the individual crop level, crop layers were overlain with the 30-m Landsat-based Irrigation Dataset (LANID)^59^ for all years included in our analysis (2008, 2011, 2013, 2016 and 2019). For the 2019 MSD layer, we used the LANID dataset for 2017 given that it was the most recent. LANID datasets were used as a mask over the original crop data layers to assign irrigated versus rainfed areas.

### Managed and unmanaged landcover

To differentiate between managed and unmanaged landcover, we used the US Geological Survey Protected Area Dataset (PADUS3)^60^ as a proxy to distinguish between “managed” and “unmanaged” lands based on land ownership, conservation status, and economic functionality of the lands. As stated in^61^, managed forest in the GCAM land system encompassed any landcover area used for annual roundwood timber production. Using the PADUS3, we attributed both public open space and voluntary private allotments to areas that were managed or unmanaged based on *‘Gap Status Codes’, which are numeric values* used to indicate conservation objectives for each parcel of land:

- Gap status code 1 – Managed for biodiversity - disturbance events proceed or are mimicked
- Gap status code 2 – Managed for biodiversity – disturbance events suppressed
- Gap status code 3 – Managed for multiple uses – subject to extractive (e.g., mining or logging) or off-road vehicle use
- Gap status code 4 – No known mandate for biodiversity protection

From these codes, GAP 3 and 4 were identified as the main areas for *managed forests*, as these areas are subject to wood harvest, whereas GAP 1 and GAP 2 are areas used exclusively for biodiversity protection. However, to cross-validate our selection, we compared the GAP 3 and 4 parcels with the USDA forest service LANDFIRE dataset (https://landfire.gov/version_download.php)^62^. We inspected forest disturbance associated with roundwood production (e.g., harvest, clearcut, other mechanical) between the years 2008–2019 and determined that these disturbances did substantially occur within all areas associated with GAP 3 and GAP 4 areas regardless of land designations at state, federal, and private levels.

We used a similar logic as managed forests to identify *managed pastures*. We selected all areas associated with *GAP 3* and only certain areas within *GAP 4*, specifically excluding recreational areas within or proximate to cities and tribal lands. Because *managed pasture* also includes areas of potential grazing, we defined used two additional sources on known grazing allotments, including the USDA Forest Service grazing dataset (https://data.fs.usda.gov/geodata/edw/datasets.php?xmlKeyword=grazing)^63^ and the USGS grazing potential dataset (https://www.usgs.gov/data/mapping-enhanced-grazing-potential-based-nawqa-wall-wall-anthropogenic-land-use-trends-nwalt)^64^.

After defining the area of managed land cover types, we then created a mask using GAP 3 and selected GAP 4 parcels and applied an overlay over the preprocessed NLCD lc classified as forest and pasture. From these overlays, we obtained the *managed forest*, *managed pasture* and *managed grassland layers*, respectively. Lastly, to identify managed areas with grazing potential, termed *managed grassland*, we overlayed the USDA Forest Service grazing allotment and USGS grazing potential dataset over the NLCD lc classified as grassland, as these areas are likely to be utilized for grazing, i.e., grazing potential^65^. All other forest, pasture, and grasslands were classified as *unmanaged* types.

### Climate zone differentiation

To accommodate climate regions in land classes, we differentiated between boreal, temperate, and tropical zones where applicable. In order to account for these ecoregional differences, we used multiple datasets, including The Nature Conservancy’s terrestrial ecoregions dataset^66^, Digital Elevation Model (DEM) data from the National Aeronautics and Space Administration’s Shuttle Radar Topography Mission (SRTM)^67^, the Koppen-Geiger Climate Classification^68^, and the North American boreal zone classifier^69^.

One challenge in directly assigning boreal ecoregions within the CONUS is that there is no consolidated boreal ecoregion in the country, except for transition zones known as hemi-boreal areas^69^. For example, one such hemi-boreal area, the subalpine ecosystem in the Rocky Mountain region, is very similar in terms of climate and vegetation to that of boreal ecosystems^70^. In our land classification system, we consider both boreal and hemi-boreal areas as boreal climate zones. Following Balliet and Hawkins^70^, we identified subalpine areas as locations with elevations above 1200 m using DEM data from the USGS SRTM: https://www.usgs.gov/centers/eros/science/usgs-eros-archive-digital-elevation-shuttle-radar-topography-mission-srtm-1. We then merged these with the North American boreal zone dataset to estimate all hemi-boreal regions in the CONUS. We then overlaid these hemi-boreal areas with the Koppen-Geiger Climate classification to determine if these regions could be classified as boreal based on temperature thresholds^70^.

Differentiating between temperate and tropical regions relied overlaying the Koppen Climate dataset on with TNC’s global ecoregions/realms to determine areas with tropical climate. We defined the following ecoregions as tropical within the CONUS based on this overlap: *Tropical and Subtropical Grasslands*, *Savannas*, and *Shrublands and Flooded Grasslands and Savannas*. All remaining areas that were not part of the boreal or tropical defined areas were classified as temperate.

### Associating MSD Landcover classes with GCAM, CLM and LUH2 land systems

**Fig. 2:**
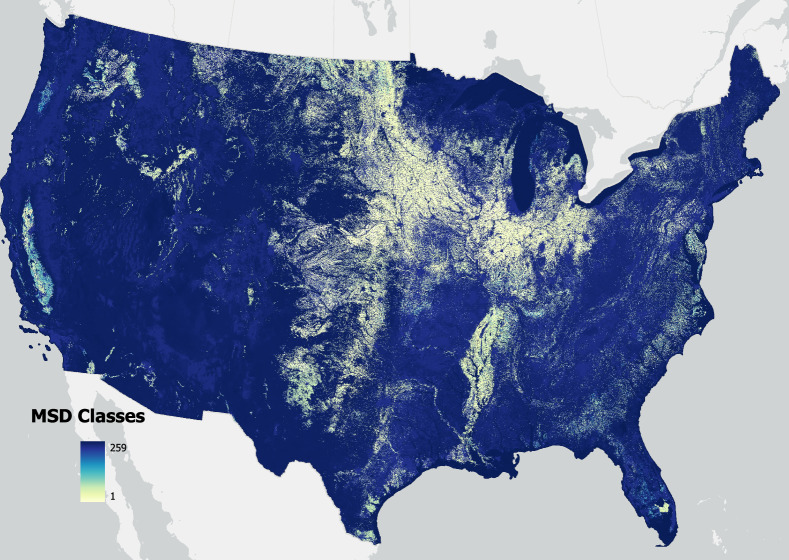
MSD landcover classes developed for the year 2008 at a 30-m resolution. Layer includes all 259 developed MSD classes that were found for that base year.

**Fig. 3:**
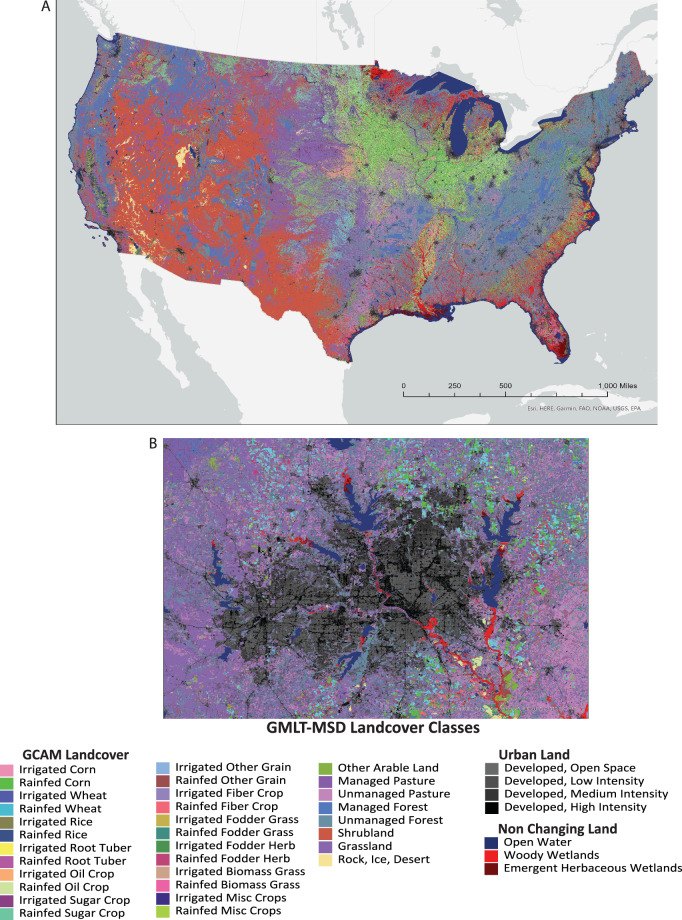
GCAM-MSD Landcover Types (GMLT) developed for the year 2008 at a 30-m resolution. (**A**) All existing GCAM landcover types within the CONUS, and (**B**) example of expanded urban classes showing the Dallas Fort Worth Area for GMLT-MSD for the year 2008.

**Fig. 4:**
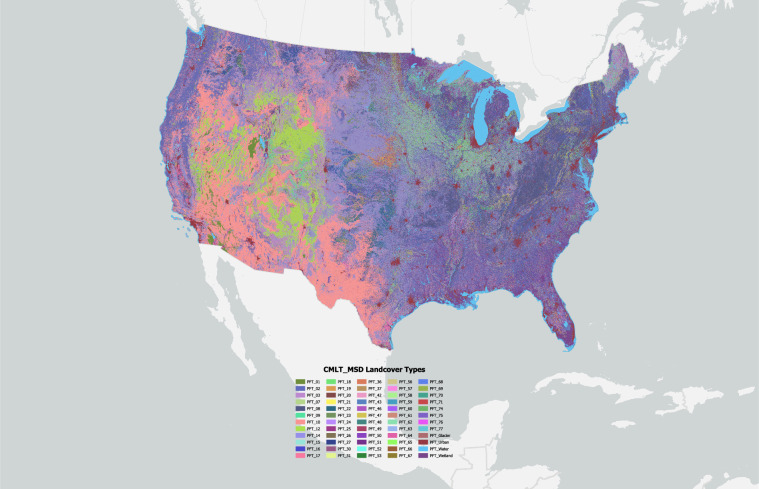
Community Land Model – MSD Landcover Types (CMLT) for the year 2008 at a 30-m resolution. Of the 79 PFTs found globally, only 63 PFTs were present in basemaps for the CONUS in addition to three static land classes for glaciers, water, wetlands, and urban areas. PFT 78 and PFT 79 were not included in the basemap because Soybean classes were exclusively temperate.

**Fig. 5:**
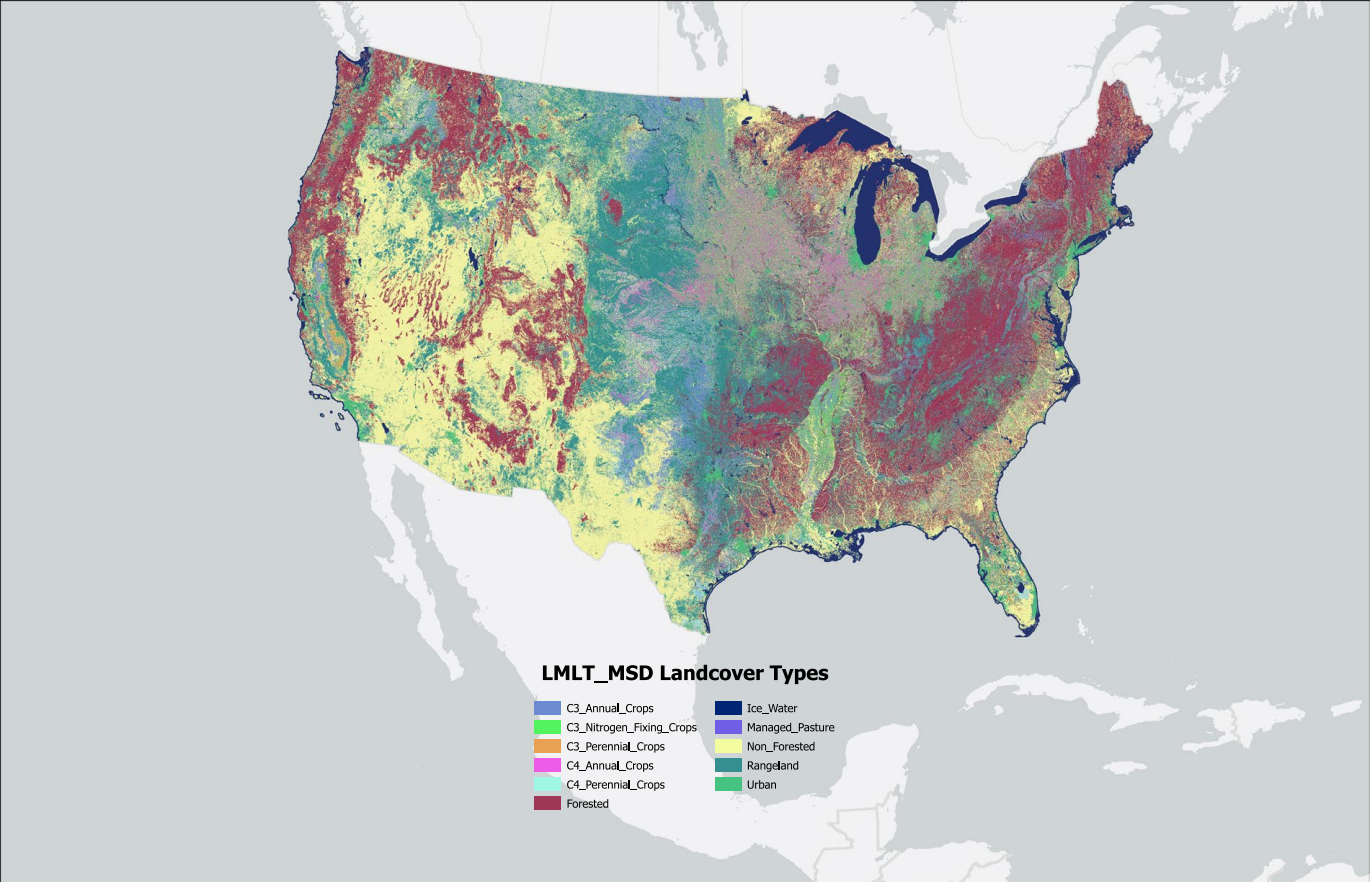
LUH2 – MSD Landcover Types (LMLT) for the year 2008 at a 30-m resolution. The *other* category includes all MSD classes that were not able to be classified into other states. Primary and secondary forested states were combined into a forested class. Non-forested landcover states were also classified into a single non-forested class.

The MSD land classification combined the specificity of empirical LULC observations (e.g., NLCD, CDL) with other spatial information on human management (e.g., irrigation, managed lands) from the geoprocessing steps described above. As such, the MSD system represents the most highly detailed and refined land classes (Fig. 2) that can be regrouped and reclassified according to compatibility with other land classification systems (Tables 1–4, Figs. 3–5). As MSD classes were regrouped to match other land classes, we re-termed these land systems as GCAM-MSD Landcover Type (GMLT) (Table 1, Fig. 3), LUH2-MSD Landcover TYPE (LMLT) (Table 2, Fig. 4), and CLM-MSD Landcover Type (CMLT) (Tables 3, 4, Fig. 5).

GMLT classes were based on the GCAM land types presented in [33]. Of the 39 original GCAM classes, only 33 were represented within the CONUS (Table 1). GCAM only includes one urban class; however, for illustration purposes, we expanded GCAM classes to include different levels of urbanization (e.g., open space, high-intensity development) found within NLCD (Figure 5). Additionally, GCAM land classes do not include wetlands or open water; therefore, these layers were included in the GMLT as *non-changing* landcover.

LUH2 states, described by^6^, were associated with MSD classes as shown in Table 2, whereas CLM PFT and MSD associations are provided in Tables 3 and 4. We followed LUH2 land state descriptions provided by^6^ to primarily identify areas classified as rangeland, forested and non-forested. For both LUH2 and CLM, we used^71^ and the FAO commodities descriptor^58^ to identify differences between C3 and C4 crops, respectively. O*pen water* and the *rock ice dessert* MSD layers were classified in the *other* category for the LMLT.

In some cases, we were unable to differentiate between CLM PFTs with MSD classes. For example, we could not differentiate between PFT 10 and PFT 11 (broadleaf evergreen shrub – temperate and broadleaf deciduous shrub – temperate) or between PFT 13 and PFT 14 (C3 Arctic and C3 Grass). We used *Temperate Shrubland* MSD classes to collectively represent PFT 10 and PFT 11, whereas both *Pasture* MSD classes and *Grassland* MSD classes represented PFTs 13 and 14 (Table 3). On the other hand, even though we found that there were PFT classes for tropical soybean (PFT 78 and PFT 79) mapped within the CONUS (based on PFT distributions), these were extremely limited in distribution. Therefore, all MSD soybean classes were associated with temperate climate.

## Data Records

The base maps consist of four different series of raster products, each representing different land classification systems for the conterminous United States: 1) a newly derived 259-class multi-sector dynamics land class system, 2) an expanded GCAM land class system (n = 39 classes), 3) a CLM land class system (n = 68 classes), and 4) an LUH2 land class system (n = 12 classes). With each series, individual raster products are provided at 30-m gridded resolution in the NAD 1983 projection for the years 2008, 2011, 2013, 2016, and 2019 based on availability of source information, primarily NLCD and CDL. Each raster represents a mutually exclusive land classification system where integer values represent only one of many land classes at each pixel location. The products can be downloaded from the MSD Live Repository^72^ under a CC BY 4.0 license. The raster products are organized into four folders, one for each land class system. The total size of the entire data is 40 GB; however, individual folders can be selected for download.

## Technical Validation

The US MSD land cover system was derived from the best available high-resolution (30-m), empirical (satellite), spatially contiguous, and comprehensive land use and land cover observation datasets, each with their own well-documented accuracy assessments^50,65^. Therefore, a validation exercise to examine the accuracy of the MSD layer would not only require significant ground-truth effort but would also be redundant with previous efforts and documentation. In contrast, we view the MSD land cover system as the standard for LULCC accuracy assessments of other land model systems within the US. Hence, a more meaningful validation consists of comparing the 30-m MSD land classification system to the pre-existing baselines, CLM and LUH2, as an assessment of spatial accuracy in those commonly used Earth land systems. This is valuable for multiple reasons. First, the CLM and LUH2 are widely accepted and used as standard LULCC systems within ESM and MSD research irrespective of their accuracy. Second, to our knowledge, no accuracy assessment of CLM and LUH2 has been accomplished in the US using the best-available satellite observations, specifically NLCD and CDL, which are generally held as the US standards for LULC mapping. Finally, such a comparison yields informative results in understanding harmonization limitations of standardized products, but also further justifies the need for an improved MSD LULCC product.

A direct comparison between MSD and the other two products, CLM and LUH2, however, is challenging due to differences in spatial resolution and data format. The land classes comprising the MSD land cover system and the products that support it are mutually exclusive land types – that is, each pixel is represented by a single land type. In contrast, the CLM and LUH2 are coarser products (0.05° and 0.25°, respectively) where multiple land types could occupy a grid cell and are each represented as percent fraction. The development of both the CLM and LUH2 required multiple assumptions of aggregating finer-scaled land processes to coarse scales^4,6^. Our comparison consisted of two main approaches to avoid biases arises from these differences: 1) evaluating overall land class association strength via pairwise comparisons between the distribution and overlap of MSD land types and the land types represented in the CLM and LUH2 products, and 2) a spatially explicit (pixel-by-pixel) comparison of the MSD land classes and the CLM and LUH2 products to understand heterogeneity in the accuracy of mapped land classes. We compared our 2008 MSD land classification with LUH2 for the same year and CLM for the year 2000. CLM 5 was chosen for the year 2000 due to data accessibility as the commonly used base year product. An important note is that CLM versions >  = 3.0 are grid-based products derived from MODIS satellite imagery^46^. Therefore, the base year for a MODIS-consistent CLM basemap is set for the year 2000. The earliest year represented in our MSD base map series is 2008 due to the availability of CDL data.

### Land class associations

The first approach assessed the overall congruency or agreement in the thematic grouping and spatial distribution of land classes between CLM and MSD classes, and between LUH2 and MSD classes. In this approach, associations are independent of thematic differences in land categories and independent of differences in numbers of classes. Even if the number of classes amongst the land classification systems did not support a 1:1 comparison, agreement in the spatial distributions of the classes could theoretically support a 100% association. For instance, if all managed and unmanaged deciduous, evergreen, and mixed forest classes from our MSD land product all occurred within the same spatial distribution of the “Forest” PFTs in CLM, this would result in a 100% association. For the CLM and LUH2 products, any one grid cell could support numerous land classes, so long as the sum of fractions across all types are 1.

Because of the difference in resolution between the MSD products and that of CLM and LUH2, we aggregated MSD classes to the 0.05° and 0.25° resolution to develop values that were compatible with CLM PFTs and LUH2 states, respectively. To support a comparison, we calculated the proportion of each MSD class occurring within each 0.05° and 0.25° grid cell using the following:

$${{MSD}}_{{ij}}=\left(\mathop{\sum }\limits_{v=1}^{n}{{MSD}}_{{jv}}\right)\times \frac{1}{n}$$

And where,

$${{MSD}}_{{jv}}\in \left\{0,1\right\}$$

In this case, the presence of an MSD class within the *v*^*th*^ 30-m grid cell is a binary outcome, and there are *n* total 30-m grid cells within each 0.05° and 0.25° grid cell. The total number of the *j*^*th*^ MSD class is summed within the *i*^*th*^ 0.05° and 0.25° grid cells and divided by the total count of 30-m grid cells within each cell. To compare unique pairwise combinations of each MSD class to each CLM PFT and LUH2 state, termed ${{MSD}}_{j}\left({C}_{k}\right)$, we used a weighted average approach, represented by the following formula:

$${{MSD}}_{j}\left({C}_{k}\right)=\frac{\mathop{\sum }\limits_{i}^{z}\left({{MSD}}_{{ij}}\times {{fC}}_{{ik}}\right)}{\mathop{\sum }\limits_{i}^{z}{{fC}}_{{ik}}}$$

Where ${{fC}}_{{ik}}$ is the fraction of the k^th^ CLM PFT found within the *i*^*th*^ 0.05° grid cell, and *z* is the total number of grid cells considered across the entire analysis. ${{MSD}}_{j}\left({C}_{k}\right)$ values represent unique proportions of land co-occurring between MSD classes and CLM PFTs and were assembled into a matrix $X$ of *j* rows of MSD classes and *k* columns of CLM PFTs.

$$X\in {{\mathbb{R}}}^{j\times k}\in \left(\frac{j}{k}\right)$$

A similar comparison between MSD classes and LUH2 states was developed using an analogous approach above where ${{fC}}_{{ik}}$ is the fraction of the k^th^ LUH2 state found within the *i*^*th*^ 0.25° grid cell. Hence, Y is a matrix representing ${{MSD}}_{j}\left({L}_{l}\right)$ proportion of areas shared between each *j*^*th*^ MSD class and *l*^*th*^ LUH2 state.

$$Y\in {{\mathbb{R}}}^{j\times l}\in \left(\frac{j}{l}\right)$$

This approach standardized all CLM PFT and LUH2 state proportions to support a viable comparison with MSD classes.

**Fig. 6:**
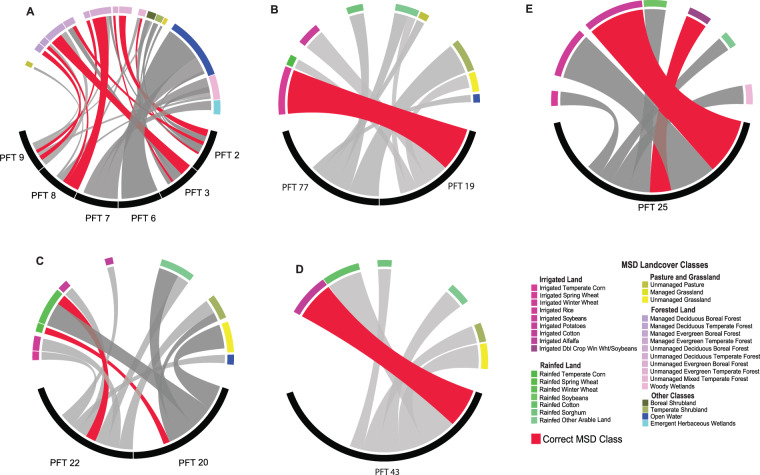
Circose diagrams comparing pairwise associations (as proportions of area) between CLM PFTs (black segments) and MSD landcover classes. Red bars depict thematically correct associations between CLM PFTs and MSD classes whereas grey bars suggest mismatches in land classes. Groups include: (**A**) Forested lands, (**B**) Irrigated Corn, (**C**) Rainfed Wheat, (**D**) Irrigated Cotton, and (**E**) Irrigated Temperate Soybeans.

**Fig. 7:**
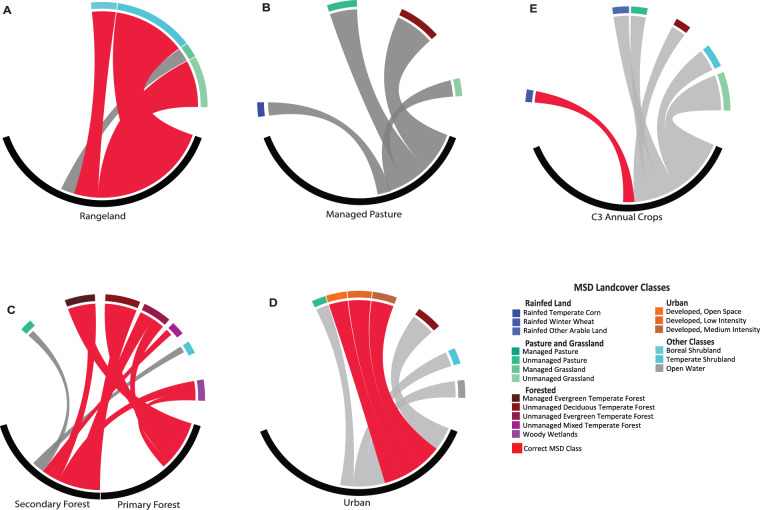
Circose diagrams comparing pairwise associations (as proportions of area) between LUH2 states and MSD landcover classes. Red bars depict thematically correct associations between LUH2 States and MSD classes whereas grey bars suggest mismatches in land classes. Groups include (**A**) Rangeland, (**B**) Managed Pasture, (**C**) Forested Land, (**D**) Urban Land, and (**E**) C3 Annual Crops.

We used the $X$ and $Y$ matrices to visualize the relationships or associations between CLM PFTs, LUH2 states and MSD classes as Circose diagrams in the Circlize package^73^ in the R programming environment (Figs. 4, 5). Circose plots visualized pairwise associations between CLM PFTs and MSD classes, and between LUH2 states and MSD classes. Relationships are represented for both CLM (Fig. 6) and LUH2 products (Fig. 7). Rare land classes representing less than 5% of the total land budget are not visually shown for simplicity.

We provide a series of examples of land class comparisons, as it was not practical to include all combinations of 259 MSD classes and CLM PFTs or LUH2 states. As seen in the figures, CLM PFTs only shared a small proportion of compatible relationships with MSD classes, where thematically correct associations are represented in red bands (Fig. 6).

We selected five land groups for comparison of CLM PFTs and MSD classes: forested land, irrigated corn, rainfed wheat, irrigated cotton, and irrigated soybean (Fig. 6A–E, respectively). In each case, all CLM PFT classes falling into each land group were selected and any MSD groups sharing associations were included. For example, there were six forested CLM PFTs (PFTs 2,3,6,7,8,9) associated with 16 MSD classes, where 50% of the association was represented by non-forested MSD classes (Fig. 6A). The strongest association was for PFT 8 (Broadleaf deciduous tree-temperate) with other compatible MSD forest classes, where 41% association was between thematically compatible classes. Similar patterns are observed for the remainder of CLM land groups.

We also compared the relationship between five LUH2 states and their respective MSD classes: Rangeland, Managed Pasture, Forested Land, Urban Land, and C3 Annual Crops (Fig. 7A–E). As found with the MSD-CLM comparison, thematically accurate associations were limited (Fig. 7), albeit more robust than CLM and MSD classes. As one example, Fig. 7B represents the relationship between the managed pasture states and MSD classes. Naturally, this state should only be represented by the managed pasture MSD class, but as demonstrated, the relationship is minimal (Fig. 7B). In total, four MSD classes accounted for 42% of the relationship with managed pasture in LUH2; however, none of these four MSD classes were classified as managed pasture in the MSD dataset. The closest representative was unmanaged pasture with only 12% relationships with LUH2. As another example, Fig. 7D examined associations between the Urban LUH2 state and MSD classes. Urban landcover from MSD classes comprised less than one third of the total area (27.29%) represented as Urban by LUH2.

### Spatial heterogeneity in mapped land classification system accuracies

To examine the spatial heterogeneity in differences between our MSD product and that of PFTs and states, we visualized mean square error (*MSE*) in compatible land group areas on a pixel-by-pixel basis. We first developed simplified and compatible groups of land classes to support a one-to-one comparison. The compatible land groupings are provided in Tables 2–4. For this comparison, we relied on the aggregation of MSD classes to the 0.05° and 0.25° resolution and ${{MSD}}_{{ij}}$ values derived for each grid in the previous analysis.

We calculated mean square error (MSE) for all compatible land class groups between the MSD and LUH2 and between MSD and CLM classes, respectively (Tables 2–4) using the following equation.

$${{{MSE}}_{i}=\frac{1}{n}\times \mathop{\sum }\limits_{j=1}^{n}({Y}_{{ij}}-{\hat{Y}}_{{ij}})}^{2}$$

**Fig. 8:**
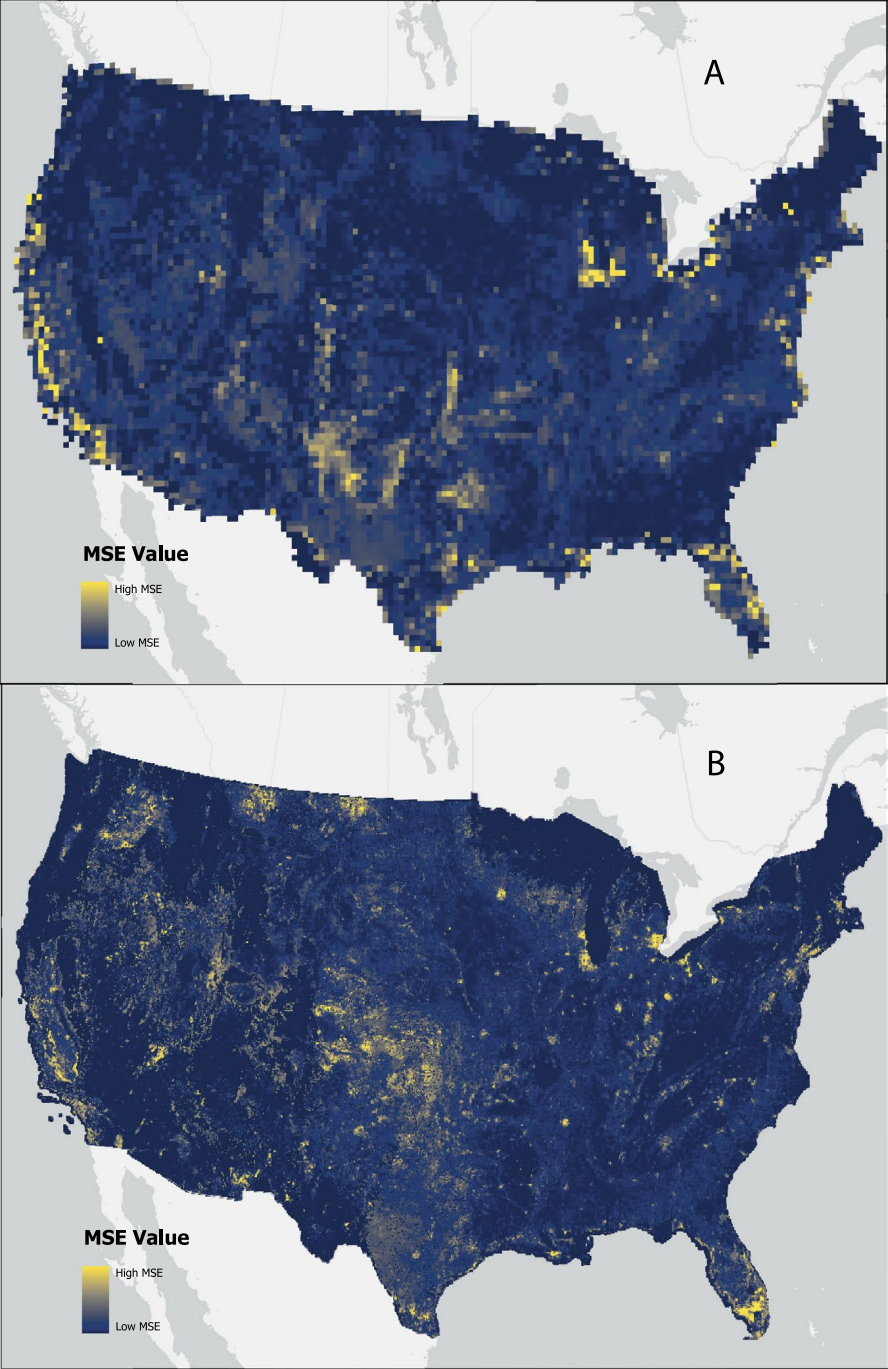
Mean square error (MSE) values comparing land groups for the (**A**) LUH2 states for 2008 and the MSD basemap for 2008, and (**B**) CLM PFTs for the year 2000 and the MSD basemap for 2008. Resolution for the LUH2 comparison is at 0.25°, whereas the CLM comparison is at 0.05°. Ranges in MSE values are based on fractionated values and are 0-0.016 for LUH2 and 0-0.018 for CLM.

Where n is the number of land class groups, *Y* are the observed proportions for the coarsened MSD groups and $\hat{Y}$ are values for CLM or LUH2 for the *i*^*th*^ grid cell and the jth land class. MSE values were then mapped across the entire CONUS (Fig. 8).

Higher MSE values for LUH2 were found in most of the central U.S., the Great Lakes region, and some segments along both coasts (Fig. 8A). Higher MSE values in these areas are an artifact of the input data representing open water areas in LUH2. Lakes and other large waterbodies are represented in an auxiliary dataset, i.e. spatial mask, for LUH2 at a coarser resolution (0.5°). For our comparison, we redistributed the values from the coarser pixels to a 0.25° resolution to ensure compatibility. Nonetheless, the discrepancy in data resolution is likely responsible for most of the higher MSE values. Other areas of higher MSE values in the central U.S., northern parts of Texas, and California were primarily due to disagreements in grazing lands and crops. Specifically in California, the highest MSE values were primarily associated with rangelands and C3 Nitrogen Fixing crops. Regions of disagreement in Northern Texas and segments of Arizona, were also due to differences in rangeland and C3 Annual Crops. These issues could be due to thematic differences in how we interpreted grazing lands, which may not approximate rangelands, which include shrublands.

In comparison, the highest MSE values for CLM were found in the Midwestern and Western U.S., but also sporadically around urban centers and the southern tip of Florida (Fig. 8B). There are multiple reasons for this pattern. Urban areas in CLM, like other non-vegetation (non-PFT) classes, are represented by separate layers, each of which serve as masks for areas in the landscape that are unchanging in future projections. In the CLM environment, the current base representation of urban lands is provided by the Spatially-Explicit, Long-term, Empirical City developmenT (SELECT) model^74,75^, where built up surfaces were derived from the Global Human Settlement Layer (GHSL)^76^. There is significant disagreement in empirical and projected global estimates of urban land, both in terms of local distribution and in aggregate regional totals^77^. These differences could be reflected in both the spatial distribution and thematic land groupings of urban lands in the MSD relative to CLM. Urban lands in our MSD product are based on the NLCD developed classes, which include open-space development, and may not agree with urban-to-rural gradients represented in the GHSL^78^. The large disagreement in Southern Florida is mostly related to the thematic differences between how wetlands are defined and mapped in the MSD layer relative to the CLM. Other disagreements between CLM and MSD are likely arising due to differences in the spatial extents of irrigation and rainfed areas and managed versus unmanaged lands, which rely on different sources between CLM and our data product. Finally, discrepancies could also be due to biases in brief time differences between CLM and MSD basemaps, 2000 vs. 2008, respectively. Heavy agriculturalized regions of the US are dynamic, characterized by routine crop rotations; therefore, even minor differences in the timing of observations behind spatial products could lead to significant spatial discrepancies.

## Usage Notes

The base maps provide a spatial product that integrates previously separate land use and land cover classification systems at a common spatial and temporal resolution. Additionally, the base maps provide thematic consistency where land use and land cover types are all relatable through a hierarchical organization of land typologies. As such, data products support more efficient regional-level integrated modeling efforts and experiments as well as more comparison and reproducibility of independent modeling efforts. The base map series can immediately support detailed assessments of land use and land cover transitions among years (2008 to 2011, 2011 to 2013, etc), but also support future regional land modeling efforts^33,79^. Most, if not all, future LULC modeling approaches require base maps as “seed” conditions for initializing model runs^33^. Beyond initial conditions, providing multiple years of base maps provides useful resources such as informing land use transition priorities, which can parameterize models, as well as some years serving as initialization whereas others can serve as validation. The resolution of the map products also supports development of hydrologic models, which also rely on accurate characterization of land use and land cover for model calibration, but also evaluating how economic growth or shifts can induce changes in land use sectors and subsequently alter hydrologic conditions.

Users of the products should be aware of the methods, assumptions, and limitations of the data. In all cases, we advise users to be aware of the methodological limitations and accuracies of the observation datasets (e.g., NLCD, CDL, MODIS) used to develop the MSD products. As is depicted in Fig. 1, NLCD products provided the spatial extent of most base information, whereas agricultural land areas received more specification from the USD CDL and MODIS. Forested land areas received more specification from climate zoning, ecoregions, and land protection status. Development of much of the land classification system was straightforward where little interpretation was required to translate some land classes (e.g., irrigated corn) into final MSD types. In other cases, however, assumptions were required to depict and map a given land type (e.g., boreal forests, managed forest). Our approach to map these land types closely followed the definitions or intentions of the pre-existing land classification systems (e.g., GCAM); obviously some assumptions were required to extend observation datasets to ensure thematic consistency in land class typologies.

## Supplementary information

Supplementary Material

## Data Availability

Customized code for processing spatial data and generating Figures 1, 6, and 7 is available in the meta-repository^80^ and on github: https://github.com/IMMM-SFA/oliver-mcmanamay_2025_scidata.
