# Supplementary material for: United States multi-sector land use and land cover base maps to support human and Earth system models
Source: Sci Data. 2025 Mar 19;12:455. doi: 10.1038/s41597-025-04713-6 (PMC11923151; doi:10.1038/s41597-025-04713-6)
Supplement: Supplementary file 1 — Supplementary Material [file 41597_2025_4713_MOESM1_ESM.docx]

Supplementary File 1. Tables

## United States Multi-Sector Dynamics land use and land cover base maps to support Human-Earth System Modeling

Jay Oliver^1^, Ryan A. McManamay^1^

^1^Department of Environmental Science, Baylor University, Waco, TX 76798

**Table S1-1.** Raw Classification for all Multisector Dynamic Layers and their corresponding CLM, LUH2 and GCAM based classification.

| **Layer Name** | **MSD Val** | **CLM Classification** | **LUH2 Classification** | **GCAM Classification** |
| --- | --- | --- | --- | --- |
| Irrigated Temperate Corn | 1 | PFT_19 | C4_Annual_Crops | Corn: Irrigated |
| Irrigated Temperate Sweet Corn | 2 | PFT_19 | C4_Annual_Crops | Corn: Irrigated |
| Irrigated Temperate Pop or Orn Corn | 3 | PFT_19 | C4_Annual_Crops | Corn: Irrigated |
| Rainfed Temperate Corn | 4 | PFT_18 | C4_Annual_Crops | Corn: Rainfed |
| Rainfed Temperate Sweet Corn | 5 | PFT_18 | C4_Annual_Crops | Corn: Rainfed |
| Rainfed Temperate Pop or Orn Corn | 6 | PFT_18 | C4_Annual_Crops | Corn: Rainfed |
| Irrigated Tropical Corn | 7 | PFT_77 | C4_Annual_Crops | Corn: Irrigated |
| Irrigated Tropical Sweet Corn | 8 | PFT_77 | C4_Annual_Crops | Corn: Irrigated |
| Irrigated Tropical Pop or Orn Corn | 9 | PFT_77 | C4_Annual_Crops | Corn: Irrigated |
| Rainfed Tropical Corn | 10 | PFT_76 | C4_Annual_Crops | Corn: Rainfed |
| Rainfed Tropical Sweet Corn | 11 | PFT_76 | C4_Annual_Crops | Corn: Rainfed |
| Rainfed Tropical Pop or Orn Corn | 12 | PFT_76 | C4_Annual_Crops | Corn: Rainfed |
| Irrigated Durum Wheat | 13 | PFT_17 | C3_Annual_Crops | Wheat: Irrigated |
| Irrigated Spring Wheat | 14 | PFT_21 | C3_Annual_Crops | Wheat: Irrigated |
| Irrigated Winter Wheat | 15 | PFT_23 | C3_Annual_Crops | Wheat: Irrigated |
| Irrigated Buckwheat | 16 | PFT_17 | C3_Annual_Crops | Wheat: Irrigated |
| Rainfed Durum Wheat | 17 | PFT_16 | C3_Annual_Crops | Wheat: Rainfed |
| Rainfed Spring Wheat | 18 | PFT_20 | C3_Annual_Crops | Wheat: Rainfed |
| Rainfed Winter Wheat | 19 | PFT_22 | C3_Annual_Crops | Wheat: Rainfed |
| Rainfed Buckwheat | 20 | PFT_16 | C3_Annual_Crops | Wheat: Rainfed |
| Irrigated Rice | 21 | PFT_63 | C3_Annual_Crops | Rice: irrigated |
| Rainfed Rice | 22 | PFT_62 | C3_Annual_Crops | Rice: rainfed |
| Irrigated Potatoes | 23 | PFT_57 | C3_Annual_Crops | Root Tuber: Irrigated |
| Irrigated Sweet Potatoes | 24 | PFT_57 | C3_Perennial_Crops | Root Tuber: Irrigated |
| Irrigated Radishes | 25 | PFT_17 | C3_Annual_Crops | Root Tuber: Irrigated |
| Rainfed Potatoes | 26 | PFT_56 | C3_Annual_Crops | Root Tuber: Rainfed |
| Rainfed Sweet Potatoes | 27 | PFT_56 | C3_Perennial_Crops | Root Tuber: Rainfed |
| Rainfed Radishes | 28 | PFT_16 | C3_Annual_Crops | Root Tuber: Rainfed |
| Irrigated Soybeans | 29 | PFT_25 | C3_Nitrogen_Fixing_Crops | Oil Crop: Irrigated |
| Irrigated Sunflower | 30 | PFT_71 | C3_Annual_Crops | Oil Crop: Irrigated |
| Irrigated Canola | 31 | PFT_17 | C3_Annual_Crops | Oil Crop: Irrigated |
| Irrigated Safflower | 32 | PFT_17 | C3_Annual_Crops | Oil Crop: Irrigated |
| Irrigated Rape Seed | 33 | PFT_61 | C3_Annual_Crops | Oil Crop: Irrigated |
| Irrigated Mustard | 34 | PFT_17 | C3_Annual_Crops | Oil Crop: Irrigated |
| Irrigated Olives | 35 | PFT_17 | C3_Perennial_Crops | Oil Crop: Irrigated |
| Rainfed Soybeans | 36 | PFT_24 | C3_Nitrogen_Fixing_Crops | Oil Crop: Rainfed |
| Rainfed Sunflower | 37 | PFT_70 | C3_Annual_Crops | Oil Crop: Rainfed |
| Rainfed Canola | 38 | PFT_16 | C3_Annual_Crops | Oil Crop: Rainfed |
| Rainfed Safflower | 39 | PFT_16 | C3_Annual_Crops | Oil Crop: Rainfed |
| Rainfed Rape Seed | 40 | PFT_60 | C3_Annual_Crops | Oil Crop: Rainfed |
| Rainfed Mustard | 41 | PFT_16 | C3_Annual_Crops | Oil Crop: Rainfed |
| Rainfed Olives | 42 | PFT_16 | C3_Perennial_Crops | Oil Crop: Rainfed |
| Irrigated Sugarbeets | 43 | PFT_69 | C3_Annual_Crops | Sugar Crop: Irrigated |
| Irrigated Sugarcane | 44 | PFT_68 | C4_Perennial_Crops | Sugar Crop: Irrigated |
| Rainfed Sugarbeets | 45 | PFT_67 | C3_Annual_Crops | Sugar Crop: Irrigated |
| Rainfed Sugarcane | 46 | PFT_66 | C4_Perennial_Crops | Sugar Crop: Rainfed |
| Irrigated Barley | 47 | PFT_27 | C3_Annual_Crops | Other Grain: Irrigated |
| Irrigated Other Small Grains | 48 | PFT_17 | C3_Annual_Crops | Other Grain: Irrigated |
| Irrigated Rye | 49 | PFT_31 | C3_Annual_Crops | Other Grain: Irrigated |
| Irrigated Oats | 50 | PFT_17 | C3_Annual_Crops | Other Grain: Irrigated |
| Irrigated Millet | 51 | PFT_53 | C4_Annual_Crops | Other Grain: Irrigated |
| Irrigated Speltz | 52 | PFT_17 | C3_Annual_Crops | Other Grain: Irrigated |
| Irrigated Triticale | 53 | PFT_17 | C3_Annual_Crops | Other Grain: Irrigated |
| Rainfed Barley | 54 | PFT_26 | C3_Annual_Crops | Other Grain: Rainfed |
| Rainfed Other Small Grains | 55 | PFT_16 | C3_Annual_Crops | Other Grain: Rainfed |
| Rainfed Rye | 56 | PFT_30 | C3_Annual_Crops | Other Grain: Rainfed |
| Rainfed Oats | 57 | PFT_16 | C3_Annual_Crops | Other Grain: Rainfed |
| Rainfed Millet | 58 | PFT_52 | C4_Annual_Crops | Other Grain: Rainfed |
| Rainfed Speltz | 59 | PFT_16 | C3_Annual_Crops | Other Grain: Rainfed |
| Rainfed Triticale | 60 | PFT_16 | C3_Annual_Crops | Other Grain: Rainfed |
| Irrigated Cotton | 61 | PFT_43 | C3_Annual_Crops | Fiber Crop: Irrigated |
| Rainfed Cotton | 62 | PFT_42 | C3_Annual_Crops | Fiber Crop: Rainfed |
| Irrigated Other Hay/Non Alfalfa | 63 | PFT_46 | C3_Annual_Crops | Fodder Grass: Irrigated |
| Rainfed Other Hay/Non Alfalfa | 64 | PFT_47 | C3_Annual_Crops | Fodder Grass: Rainfed |
| Irrigated Sorghum | 65 | PFT_65 | C4_Perennial_Crops | Fodder Herb: Irrigated |
| Irrigated Alfalfa | 66 | PFT_17 | C3_Nitrogen_Fixing_Crops | Fodder Herb: Irrigated |
| Irrigated Clover/Wildflowers | 67 | PFT_17 | C3_Annual_Crops | Fodder Herb: Irrigated |
| Irrigated Vetch | 68 | PFT_17 | C3_Annual_Crops | Fodder Herb: Irrigated |
| Rainfed Sorghum | 69 | PFT_64 | C4_Perennial_Crops | Fodder Herb: Rainfed |
| Rainfed Alfalfa | 70 | PFT_16 | C3_Nitrogen_Fixing_Crops | Fodder Herb: Rainfed |
| Rainfed Clover/Wildflowers | 71 | PFT_16 | C3_Annual_Crops | Fodder Herb: Rainfed |
| Rainfed Vetch | 72 | PFT_16 | C3_Annual_Crops | Fodder Herb: Rainfed |
| Irrigated Camelina | 73 | PFT_17 | C3_Annual_Crops | Biomass Grass: Irrigated |
| Irrigated Switchgrass | 74 | PFT_75 | C3_Perennial_Crops | Biomass Grass: Irrigated |
| Rainfed Camelina | 75 | PFT_16 | C3_Annual_Crops | Biomass Grass: Rainfed |
| Rainfed Switchgrass | 76 | PFT_74 | C3_Perennial_Crops | Biomass Grass: Rainfed |
| Irrigated Peanuts | 77 | PFT_51 | C3_Nitrogen_Fixing_Crops | Misc-Crop: Irrigated |
| Irrigated Tobacco | 78 | PFT_17 | C3_Annual_Crops | Misc-Crop: Irrigated |
| Irrigated Mint | 79 | PFT_17 | C3_Perennial_Crops | Misc-Crop: Irrigated |
| Irrigated Dbl Crop Win Wht/Soybeans | 80 | PFT_23 | C3_Nitrogen_Fixing_Crops | Misc-Crop: Irrigated |
| Irrigated Flaxseed | 81 | PFT_17 | C3_Annual_Crops | Misc-Crop: Irrigated |
| Irrigated Dry Beans | 82 | PFT_59 | C3_Nitrogen_Fixing_Crops | Misc-Crop: Irrigated |
| Irrigated Other Crops | 83 | PFT_17 | C3_Annual_Crops | Misc-Crop: Irrigated |
| Irrigated Misc Vegs & Fruits | 84 | PFT_17 | C3_Annual_Crops | Misc-Crop: Irrigated |
| Irrigated Watermelons | 85 | PFT_17 | C3_Annual_Crops | Misc-Crop: Irrigated |
| Irrigated Onions | 86 | PFT_17 | C3_Perennial_Crops | Misc-Crop: Irrigated |
| Irrigated Cucumbers | 87 | PFT_17 | C3_Annual_Crops | Misc-Crop: Irrigated |
| Irrigated Chick Peas | 88 | PFT_59 | C3_Annual_Crops | Misc-Crop: Irrigated |
| Irrigated Lentils | 89 | PFT_59 | C3_Nitrogen_Fixing_Crops | Misc-Crop: Irrigated |
| Irrigated Peas | 90 | PFT_59 | C3_Nitrogen_Fixing_Crops | Misc-Crop: Irrigated |
| Irrigated Tomatoes | 91 | PFT_17 | C3_Annual_Crops | Misc-Crop: Irrigated |
| Irrigated Caneberries | 92 | PFT_17 | C3_Perennial_Crops | Misc-Crop: Irrigated |
| Irrigated Hops | 93 | PFT_17 | C3_Perennial_Crops | Misc-Crop: Irrigated |
| Irrigated Herbs | 94 | PFT_17 | C3_Perennial_Crops | Misc-Crop: Irrigated |
| Irrigated Sod/Grass Seed | 95 | PFT_17 | C3_Perennial_Crops | Misc-Crop: Irrigated |
| Irrigated Cherries | 96 | PFT_17 | C3_Perennial_Crops | Misc-Crop: Irrigated |
| Irrigated Peaches | 97 | PFT_17 | C3_Perennial_Crops | Misc-Crop: Irrigated |
| Irrigated Apples | 98 | PFT_17 | C3_Perennial_Crops | Misc-Crop: Irrigated |
| Irrigated Grapes | 99 | PFT_49 | C3_Perennial_Crops | Misc-Crop: Irrigated |
| Irrigated Christmas Trees | 100 | PFT_17 | C3_Annual_Crops | Misc-Crop: Irrigated |
| Irrigated Other Tree Crops | 101 | PFT_17 | C3_Annual_Crops | Misc-Crop: Irrigated |
| Irrigated Citrus | 102 | PFT_37 | C3_Perennial_Crops | Misc-Crop: Irrigated |
| Irrigated Pecans | 103 | PFT_17 | C3_Perennial_Crops | Misc-Crop: Irrigated |
| Irrigated Almonds | 104 | PFT_17 | C3_Perennial_Crops | Misc-Crop: Irrigated |
| Irrigated Walnuts | 105 | PFT_17 | C3_Perennial_Crops | Misc-Crop: Irrigated |
| Irrigated Pears | 106 | PFT_17 | C3_Perennial_Crops | Misc-Crop: Irrigated |
| Irrigated Pistachios | 107 | PFT_17 | C3_Perennial_Crops | Misc-Crop: Irrigated |
| Irrigated Carrots | 108 | PFT_17 | C3_Annual_Crops | Misc-Crop: Irrigated |
| Irrigated Asparagus | 109 | PFT_17 | C3_Perennial_Crops | Misc-Crop: Irrigated |
| Irrigated Garlic | 110 | PFT_17 | C3_Perennial_Crops | Misc-Crop: Irrigated |
| Irrigated Cantaloupes | 111 | PFT_17 | C3_Annual_Crops | Misc-Crop: Irrigated |
| Irrigated Prunes | 112 | PFT_17 | C3_Annual_Crops | Misc-Crop: Irrigated |
| Irrigated Oranges | 113 | PFT_37 | C3_Perennial_Crops | Misc-Crop: Irrigated |
| Irrigated Honeydew Melons | 114 | PFT_17 | C3_Annual_Crops | Misc-Crop: Irrigated |
| Irrigated Broccoli | 115 | PFT_17 | C3_Perennial_Crops | Misc-Crop: Irrigated |
| Irrigated Avocados | 116 | PFT_17 | C3_Perennial_Crops | Misc-Crop: Irrigated |
| Irrigated Peppers | 117 | PFT_17 | C3_Perennial_Crops | Misc-Crop: Irrigated |
| Irrigated Pomegranates | 118 | PFT_17 | C3_Perennial_Crops | Misc-Crop: Irrigated |
| Irrigated Nectarines | 119 | PFT_37 | C3_Perennial_Crops | Misc-Crop: Irrigated |
| Irrigated Greens | 120 | PFT_17 | C3_Annual_Crops | Misc-Crop: Irrigated |
| Irrigated Plums | 121 | PFT_17 | C3_Perennial_Crops | Misc-Crop: Irrigated |
| Irrigated Strawberries | 122 | PFT_17 | C3_Perennial_Crops | Misc-Crop: Irrigated |
| Irrigated Squash | 123 | PFT_17 | C3_Annual_Crops | Misc-Crop: Irrigated |
| Irrigated Apricots | 124 | PFT_17 | C3_Perennial_Crops | Misc-Crop: Irrigated |
| Irrigated Dbl Crop Win Wht/Corn | 125 | PFT_23 | C3_Annual_Crops | Misc-Crop: Irrigated |
| Irrigated Dbl Crop Oats/Corn | 126 | PFT_17 | C3_Annual_Crops | Misc-Crop: Irrigated |
| Irrigated Lettuce | 127 | PFT_17 | C3_Annual_Crops | Misc-Crop: Irrigated |
| Irrigated Dbl Crop Triticale/Corn | 128 | PFT_17 | C3_Annual_Crops | Misc-Crop: Irrigated |
| Irrigated Pumpkins | 129 | PFT_17 | C3_Annual_Crops | Misc-Crop: Irrigated |
| Irrigated Dbl Crop Lettuce/Durum Wht | 130 | PFT_17 | C3_Annual_Crops | Misc-Crop: Irrigated |
| Irrigated Dbl Crop Lettuce/Cantaloupe | 131 | PFT_17 | C3_Annual_Crops | Misc-Crop: Irrigated |
| Irrigated Dbl Crop Lettuce/Cotton | 132 | PFT_17 | C3_Annual_Crops | Misc-Crop: Irrigated |
| Irrigated Dbl Crop Lettuce/Barley | 133 | PFT_17 | C3_Annual_Crops | Misc-Crop: Irrigated |
| Irrigated Dbl Crop Durum Wht/Sorghum | 134 | PFT_17 | C3_Annual_Crops | Misc-Crop: Irrigated |
| Irrigated Dbl Crop Barley/Sorghum | 135 | PFT_27 | C4_Annual_Crops | Misc-Crop: Irrigated |
| Irrigated Dbl Crop Win Wht/Sorghum | 136 | PFT_23 | C3_Annual_Crops | Misc-Crop: Irrigated |
| Irrigated Dbl Crop Barley/Corn | 137 | PFT_27 | C4_Annual_Crops | Misc-Crop: Irrigated |
| Irrigated Dbl Crop Win Wht/Cotton | 138 | PFT_23 | C3_Annual_Crops | Misc-Crop: Irrigated |
| Irrigated Dbl Crop Soybeans/Cotton | 139 | PFT_25 | C3_Nitrogen_Fixing_Crops | Misc-Crop: Irrigated |
| Irrigated Dbl Crop Soybeans/Oats | 140 | PFT_25 | C3_Nitrogen_Fixing_Crops | Misc-Crop: Irrigated |
| Irrigated Dbl Crop Corn/Soybeans | 141 | PFT_19 | C3_Nitrogen_Fixing_Crops | Misc-Crop: Irrigated |
| Irrigated Blueberries | 142 | PFT_17 | C3_Perennial_Crops | Misc-Crop: Irrigated |
| Irrigated Cabbage | 143 | PFT_17 | C3_Annual_Crops | Misc-Crop: Irrigated |
| Irrigated Cauliflower | 144 | PFT_17 | C3_Annual_Crops | Misc-Crop: Irrigated |
| Irrigated Celery | 145 | PFT_17 | C3_Annual_Crops | Misc-Crop: Irrigated |
| Irrigated Turnips | 146 | PFT_17 | C3_Annual_Crops | Misc-Crop: Irrigated |
| Irrigated Eggplants | 147 | PFT_17 | C3_Annual_Crops | Misc-Crop: Irrigated |
| Irrigated Gourds | 148 | PFT_17 | C3_Annual_Crops | Misc-Crop: Irrigated |
| Irrigated Cranberries | 149 | PFT_17 | C3_Perennial_Crops | Misc-Crop: Irrigated |
| Irrigated Dbl Crop Barley/Soybeans | 150 | PFT_27 | C3_Nitrogen_Fixing_Crops | Misc-Crop: Irrigated |
| Rainfed Peanuts | 151 | PFT_50 | C3_Nitrogen_Fixing_Crops | Misc-Crop: Rainfed |
| Rainfed Tobacco | 152 | PFT_16 | C3_Annual_Crops | Misc-Crop: Rainfed |
| Rainfed Mint | 153 | PFT_16 | C3_Perennial_Crops | Misc-Crop: Rainfed |
| Rainfed Dbl Crop Win Wht/Soybeans | 154 | PFT_22 | C3_Nitrogen_Fixing_Crops | Misc-Crop: Rainfed |
| Rainfed Flaxseed | 155 | PFT_16 | C3_Annual_Crops | Misc-Crop: Rainfed |
| Rainfed Dry Beans | 156 | PFT_58 | C3_Nitrogen_Fixing_Crops | Misc-Crop: Rainfed |
| Rainfed Other Crops | 157 | PFT_16 | C3_Annual_Crops | Misc-Crop: Rainfed |
| Rainfed Misc Vegs & Fruits | 158 | PFT_16 | C3_Annual_Crops | Misc-Crop: Rainfed |
| Rainfed Watermelons | 159 | PFT_16 | C3_Annual_Crops | Misc-Crop: Rainfed |
| Rainfed Onions | 160 | PFT_16 | C3_Perennial_Crops | Misc-Crop: Rainfed |
| Rainfed Cucumbers | 161 | PFT_16 | C3_Annual_Crops | Misc-Crop: Rainfed |
| Rainfed Chick Peas | 162 | PFT_58 | C3_Annual_Crops | Misc-Crop: Rainfed |
| Rainfed Lentils | 163 | PFT_58 | C3_Nitrogen_Fixing_Crops | Misc-Crop: Rainfed |
| Rainfed Peas | 164 | PFT_58 | C3_Nitrogen_Fixing_Crops | Misc-Crop: Rainfed |
| Rainfed Tomatoes | 165 | PFT_16 | C3_Annual_Crops | Misc-Crop: Rainfed |
| Rainfed Caneberries | 166 | PFT_16 | C3_Perennial_Crops | Misc-Crop: Rainfed |
| Rainfed Hops | 167 | PFT_16 | C3_Perennial_Crops | Misc-Crop: Rainfed |
| Rainfed Herbs | 168 | PFT_16 | C3_Perennial_Crops | Misc-Crop: Rainfed |
| Rainfed Sod/Grass Seed | 169 | PFT_16 | C3_Perennial_Crops | Misc-Crop: Rainfed |
| Rainfed Cherries | 170 | PFT_16 | C3_Perennial_Crops | Misc-Crop: Rainfed |
| Rainfed Peaches | 171 | PFT_16 | C3_Perennial_Crops | Misc-Crop: Rainfed |
| Rainfed Apples | 172 | PFT_16 | C3_Perennial_Crops | Misc-Crop: Rainfed |
| Rainfed Grapes | 173 | PFT_48 | C3_Perennial_Crops | Misc-Crop: Rainfed |
| Rainfed Christmas Trees | 174 | PFT_16 | C3_Annual_Crops | Misc-Crop: Rainfed |
| Rainfed Other Tree Crops | 175 | PFT_16 | C3_Annual_Crops | Misc-Crop: Rainfed |
| Rainfed Citrus | 176 | PFT_36 | C3_Perennial_Crops | Misc-Crop: Rainfed |
| Rainfed Pecans | 177 | PFT_16 | C3_Perennial_Crops | Misc-Crop: Rainfed |
| Rainfed Almonds | 178 | PFT_16 | C3_Perennial_Crops | Misc-Crop: Rainfed |
| Rainfed Walnuts | 179 | PFT_16 | C3_Perennial_Crops | Misc-Crop: Rainfed |
| Rainfed Pears | 180 | PFT_16 | C3_Perennial_Crops | Misc-Crop: Rainfed |
| Rainfed Pistachios | 181 | PFT_16 | C3_Perennial_Crops | Misc-Crop: Rainfed |
| Rainfed Carrots | 182 | PFT_16 | C3_Annual_Crops | Misc-Crop: Rainfed |
| Rainfed Asparagus | 183 | PFT_16 | C3_Perennial_Crops | Misc-Crop: Rainfed |
| Rainfed Garlic | 184 | PFT_16 | C3_Perennial_Crops | Misc-Crop: Rainfed |
| Rainfed Cantaloupes | 185 | PFT_16 | C3_Annual_Crops | Misc-Crop: Rainfed |
| Rainfed Prunes | 186 | PFT_16 | C3_Annual_Crops | Misc-Crop: Rainfed |
| Rainfed Oranges | 187 | PFT_36 | C3_Perennial_Crops | Misc-Crop: Rainfed |
| Rainfed Honeydew Melons | 188 | PFT_16 | C3_Annual_Crops | Misc-Crop: Rainfed |
| Rainfed Broccoli | 189 | PFT_16 | C3_Perennial_Crops | Misc-Crop: Rainfed |
| Rainfed Avocados | 190 | PFT_17 | C3_Perennial_Crops | Misc-Crop: Rainfed |
| Rainfed Peppers | 191 | PFT_16 | C3_Perennial_Crops | Misc-Crop: Rainfed |
| Rainfed Pomegranates | 192 | PFT_16 | C3_Perennial_Crops | Misc-Crop: Rainfed |
| Rainfed Nectarines | 193 | PFT_36 | C3_Perennial_Crops | Misc-Crop: Rainfed |
| Rainfed Greens | 194 | PFT_16 | C3_Annual_Crops | Misc-Crop: Rainfed |
| Rainfed Plums | 195 | PFT_16 | C3_Perennial_Crops | Misc-Crop: Rainfed |
| Rainfed Strawberries | 196 | PFT_16 | C3_Perennial_Crops | Misc-Crop: Rainfed |
| Rainfed Squash | 197 | PFT_16 | C3_Annual_Crops | Misc-Crop: Rainfed |
| Rainfed Apricots | 198 | PFT_16 | C3_Perennial_Crops | Misc-Crop: Rainfed |
| Rainfed Dbl Crop Win Wht/Corn | 199 | PFT_22 | C3_Annual_Crops | Misc-Crop: Rainfed |
| Rainfed Dbl Crop Oats/Corn | 200 | PFT_16 | C3_Annual_Crops | Misc-Crop: Rainfed |
| Rainfed Lettuce | 201 | PFT_16 | C3_Annual_Crops | Misc-Crop: Rainfed |
| Rainfed Dbl Crop Triticale/Corn | 202 | PFT_16 | C3_Annual_Crops | Misc-Crop: Rainfed |
| Rainfed Pumpkins | 203 | PFT_16 | C3_Annual_Crops | Misc-Crop: Rainfed |
| Rainfed Dbl Crop Lettuce/Durum Wht | 204 | PFT_16 | C3_Annual_Crops | Misc-Crop: Rainfed |
| Rainfed Dbl Crop Lettuce/Cantaloupe | 205 | PFT_16 | C3_Annual_Crops | Misc-Crop: Rainfed |
| Rainfed Dbl Crop Lettuce/Cotton | 206 | PFT_16 | C3_Annual_Crops | Misc-Crop: Rainfed |
| Rainfed Dbl Crop Lettuce/Barley | 207 | PFT_16 | C3_Annual_Crops | Misc-Crop: Rainfed |
| Rainfed Dbl Crop Durum Wht/Sorghum | 208 | PFT_16 | C3_Annual_Crops | Misc-Crop: Rainfed |
| Rainfed Dbl Crop Barley/Sorghum | 209 | PFT_26 | C4_Annual_Crops | Misc-Crop: Rainfed |
| Rainfed Dbl Crop Win Wht/Sorghum | 210 | PFT_22 | C3_Annual_Crops | Misc-Crop: Rainfed |
| Rainfed Dbl Crop Barley/Corn | 211 | PFT_26 | C4_Annual_Crops | Misc-Crop: Rainfed |
| Rainfed Dbl Crop Win Wht/Cotton | 212 | PFT_22 | C3_Annual_Crops | Misc-Crop: Rainfed |
| Rainfed Dbl Crop Soybeans/Cotton | 213 | PFT_24 | C3_Nitrogen_Fixing_Crops | Misc-Crop: Rainfed |
| Rainfed Dbl Crop Soybeans/Oats | 214 | PFT_24 | C3_Nitrogen_Fixing_Crops | Misc-Crop: Rainfed |
| Rainfed Dbl Crop Corn/Soybeans | 215 | PFT_18 | C3_Nitrogen_Fixing_Crops | Misc-Crop: Rainfed |
| Rainfed Blueberries | 216 | PFT_16 | C3_Perennial_Crops | Misc-Crop: Rainfed |
| Rainfed Cabbage | 217 | PFT_16 | C3_Annual_Crops | Misc-Crop: Rainfed |
| Rainfed Cauliflower | 218 | PFT_16 | C3_Annual_Crops | Misc-Crop: Rainfed |
| Rainfed Celery | 219 | PFT_16 | C3_Annual_Crops | Misc-Crop: Rainfed |
| Rainfed Turnips | 220 | PFT_16 | C3_Annual_Crops | Misc-Crop: Rainfed |
| Rainfed Eggplants | 221 | PFT_16 | C3_Annual_Crops | Misc-Crop: Rainfed |
| Rainfed Gourds | 222 | PFT_16 | C3_Annual_Crops | Misc-Crop: Rainfed |
| Rainfed Cranberries | 223 | PFT_16 | C3_Perennial_Crops | Misc-Crop: Rainfed |
| Rainfed Dbl Crop Barley/Soybeans | 224 | PFT_26 | C3_Nitrogen_Fixing_Crops | Misc-Crop: Rainfed |
| Irrigated Other Arable Land | 225 | PFT_15 | Non_Forested_2009 | Other Arable Land |
| Rainfed Other Arable Land | 226 | PFT_16 | Non_Forested_2009 | Other Arable Land |
| Managed Pasture | 227 | PFT_14 | Managed_Pasture | Managed Pasture |
| Unmanaged Pasture | 228 | PFT_14 | Rangeland | Unmanaged Pasture |
| Developed, Open Space | 229 | PFT_01 | Urban | Developed |
| Developed, Low Intensity | 230 | PFT_01 | Urban | Developed |
| Developed, Medium Intensity | 231 | PFT_01 | Urban | Developed |
| Developed, High Intensity | 232 | PFT_01 | Urban | Developed |
| Managed Deciduous Boreal Forest | 233 | PFT_09 | Forested | Managed Forest |
| Managed Deciduous Temperate Forest | 234 | PFT_08 | Forested | Managed Forest |
| Managed Deciduous Tropical Forest | 235 | PFT_07 | Forested | Managed Forest |
| Managed Evergreen Boreal Forest | 236 | PFT_03 | Forested | Managed Forest |
| Managed Evergreen Temperate Forest | 237 | PFT_02 | Forested | Managed Forest |
| Managed Evergreen Tropcial Forest | 238 | PFT_07 | Forested | Managed Forest |
| Managed Mixed Boreal Forest | 239 | PFT_03 | Forested | Managed Forest |
| Managed Mixed Temperate Forest | 240 | PFT_02 | Forested | Managed Forest |
| Managed Mixed Tropical Forest | 241 | PFT_07 | Forested | Managed Forest |
| Unmanaged Deciduous Boreal Forest | 242 | PFT_09 | Forested | Unmanaged Forest |
| Unmanaged Deciduous Temperate Forest | 243 | PFT_08 | Forested | Unmanaged Forest |
| Unmanaged Deciduous Tropical Forest | 244 | PFT_07 | Forested | Unmanaged Forest |
| Unmanaged Evergreen Boreal Forest | 245 | PFT_03 | Forested | Unmanaged Forest |
| Unmanaged Evergreen Temperate Forest | 246 | PFT_02 | Forested | Unmanaged Forest |
| Unmanaged Evergreen Tropcial Forest | 247 | PFT_07 | Forested | Unmanaged Forest |
| Unmanaged Mixed Boreal Forest | 248 | PFT_03 | Forested | Unmanaged Forest |
| Unmanaged Mixed Temperate Forest | 249 | PFT_02 | Forested | Unmanaged Forest |
| Unmanaged Mixed Tropical Forest | 250 | PFT_07 | Forested | Unmanaged Forest |
| Boreal shrubland | 251 | PFT_12 | Non_Forested | Shrubland |
| Temperate Shrubland | 252 | PFT_10 | Non_Forested | Shrubland |
| Managed Grassland | 253 | PFT_14 | Rangeland | Managed Pasture |
| Unmanaged Grassland | 254 | PFT_14 | Rangeland | Grassland |
| Perennial Snow/Ice | 255 | PFT_Glacier | Ice_Water | Rock, Ice, Desert |
| Barren Land | 256 | PFT_01 | Non_Forested | Rock, Ice, Desert |
| Open Water | 257 | PFT_Water | Ice_Water | Open Water |
| Woody Wetlands | 258 | PFT_Wetland | Forested | Woody Wetlands |
| Emergent Herbaceous Wetlands | 259 | PFT_Wetland | Non_Forested | Emergent Herbaceous Wetlands |
